# Fatty acid oxidation impairment in diabetic kidney disease: a bidirectional feedback network from mitochondrial quality control to systemic regulation

**DOI:** 10.3389/fendo.2026.1968762

**Published:** 2026-10-19

**Authors:** Yuanxin Jiang, Wudi Zhang, Danmai Zhao, Zhe Chen, Zhonghong Yan

**Affiliations:** Heilongjiang University of Chinese Medicine, Harbin, China

**Keywords:** diabetic kidney disease, endoplasmic reticulum stress, fatty acid oxidation, ferroptosis, gut microbiota, metabolic reprogramming, mitochondrial quality control

## Abstract

Diabetic kidney disease (DKD) is a major microvascular complication of diabetes with complex metabolic pathogenesis and limited therapeutic options. Defective fatty acid oxidation (FAO) and consequent lipotoxicity are central to renal fibrosis, but their systematic integration with other DKD pathologies remains incomplete. This Review examines the molecular links between FAO impairment and mitochondrial quality control, metabolic reprogramming, ferroptosis, ER stress, and gut dysbiosis. Based on current evidence, we propose that in diabetic kidney disease, defective fatty acid oxidation and impaired mitochondrial quality control form a self-amplifying bidirectional positive feedback loop. Hyperglycemia and lipotoxicity suppress PGC-1α-mediated mitochondrial biogenesis, promote excessive mitochondrial fission, and lead to impaired PINK1/Parkin-dependent mitophagy, thereby reducing fatty acid oxidation efficiency. The resulting ATP deficiency and lipid accumulation, in turn, elevate mitochondrial ROS levels and further exacerbate mitochondrial quality control dysregulation. This energy crisis then drives aberrant lipid accumulation, compensatory glycolytic enhancement, and blocked branched-chain amino acid catabolism, and amplifies injury signals through pathways such as ferroptosis and ER stress, ultimately establishing a systemic vicious cycle via the gut–kidney axis that promotes tubular injury and interstitial fibrosis. We also summarize emerging FAO-targeted interventions, including natural compounds, modern drugs, Chinese patent medicines, and lifestyle strategies. Although FAO impairment is now recognized as a key node in DKD progression, most evidence remains preclinical. Future integration of multi-omics and dynamic metabolic tracing is needed to establish causality and stage-specific roles, while dissecting the FAO regulatory architecture across renal cell types may offer new early diagnostic and therapeutic targets.

## 1 Introduction

Diabetic kidney disease (DKD) is among the most severe microvascular complications of diabetes, affecting approximately 30% of patients with type 1 diabetes and 40% of those with type 2 diabetes (1). DKD is also recognized as the leading global cause of chronic kidney disease and end-stage renal disease (ESRD) (2). Long-standing hyperglycaemia, hypertension and protracted disease duration are the principal risk factors for DKD (3). The early pathological changes of DKD are dominated by persistent albuminuria and glomerular hyperfiltration, which are followed by structural and functional alterations of the glomerulus, including mesangial matrix expansion, basement membrane thickening and increased extracellular matrix (ECM) deposition. These changes lead to renal hypertrophy, podocyte injury and endothelial dysfunction, and subsequently to glomerulosclerosis and tubulointerstitial fibrosis, culminating in ESRD (4, 5). In addition, metabolic abnormalities such as fatty acid accumulation, carbonyl stress and oxidative stress are closely associated with DKD progression (4). Among these, lipotoxicity arising from defective fatty acid oxidation is a key factor driving renal fibrosis (6). Current pharmacological management of DKD relies primarily on renin–angiotensin–aldosterone system inhibitors (RASi), sodium–glucose cotransporter 2 (SGLT2) inhibitors, glucagon-like peptide-1 receptor agonists (GLP-1RAs) and non-steroidal mineralocorticoid receptor antagonists (NS-MRAs) (1). Nevertheless, available treatments still fail to effectively arrest DKD progression, and their long-term clinical use is associated with adverse effects such as hyperkalaemia and urinary tract infection. There is therefore an urgent need for new preventive and therapeutic strategies (7, 8).

Fatty acid oxidation (FAO, also known as β-oxidation) occurs predominantly within mitochondria and represents the core pathway through which fatty acids are catabolized to supply energy (8). The kidney is one of the most energy-demanding organs in the human body, with a mitochondrial abundance and oxygen consumption second only to the heart. Large amounts of adenosine triphosphate (ATP) are required to sustain physiological processes including nutrient reabsorption, acid–base homeostasis, and the regulation of electrolyte and fluid balance (9). ATP generation in the kidney displays marked cell-type specificity: podocytes, glomerular endothelial cells and mesangial cells rely mainly on glucose metabolism, whereas proximal tubular epithelial cells, the most energy-consuming cell population in the kidney, depend predominantly on FAO for ATP production (10, 11). When oxygen supply is insufficient and FAO is consequently impeded, ATP production falls short, and incompletely degraded fatty acids generate lipid intermediates that provoke lipotoxicity, driving renal fibrosis and DKD progression (12). FAO is thus essential for maintaining normal renal physiology and participates in virtually all DKD-related pathological processes. Preserving FAO homeostasis has accordingly emerged as a core target for DKD intervention.

Accumulating evidence indicates that FAO impairment is not an isolated metabolic event but rather forms a complex interaction network with mitochondrial quality control disturbance, substrate metabolic reprogramming, ferroptosis, ER stress and gut dysbiosis, all of which are key pathological processes in DKD. Against this background, this review proposes that in DKD, impaired mitochondrial quality control and defective FAO form a bidirectional loop. On the one hand, suppression of PGC-1α-mediated mitochondrial biogenesis, activation of excessive mitochondrial fission, and inhibition of PINK1/Parkin-mediated mitophagy act in concert to cause a sustained decline in FAO efficiency. On the other hand, reduced FAO efficiency further exacerbates mitochondrial quality control dysregulation. The resulting energy crisis further drives metabolic reprogramming of lipids, glucose, and amino acids, and amplifies injury signals through pathological pathways such as ferroptosis and ER stress, ultimately forming a systemic vicious cycle via the gut–kidney axis that promotes tubular injury and interstitial fibrosis. Here we systematically delineate the molecular links between FAO impairment and the aforementioned pathological processes, summarize the latest FAO-targeted therapeutic strategies for DKD, and offer perspectives on current research limitations and future directions, with the aim of providing new conceptual angles and a theoretical basis for mechanistic studies and clinical management of DKD.

## 2 Fatty acid oxidation under physiological conditions

Fatty acids are key components of triglycerides and are conventionally classified according to hydrocarbon chain length into short-chain (SCFAs), medium-chain (MCFAs), and long-chain fatty acids (LCFAs); free fatty acids represent their non-esterified form (13). Oxidative degradation of fatty acids takes place mainly in mitochondria, with a minor fraction occurring in peroxisomes; this process, termed β-oxidation, is the dominant mode of mitochondrial fatty acid metabolism (**Figure 1**) (14). Before being transported into the mitochondrial matrix, free fatty acids are first activated to acyl-CoA by acyl-CoA synthetases (ACSs) and are then shuttled across the mitochondrial membranes by carnitine palmitoyltransferases (CPTs) (15). The CPT family comprises four distinct proteins, CPT1A, CPT1B, CPT1C and CPT2. CPT1 family members are finely regulated by malonyl-CoA, whose intracellular level directly reflects the cellular nutritional state (16). CPT1 converts acyl-CoA to acylcarnitine, which crosses the mitochondrial inner membrane and is then reconverted to acyl-CoA by CPT2, thereby initiating β-oxidation (17). The kidney is a highly metabolic organ that depends on large quantities of ATP to sustain its filtration and reabsorption functions, and mitochondrial FAO is its principal energy source (18).

**Figure 1:**
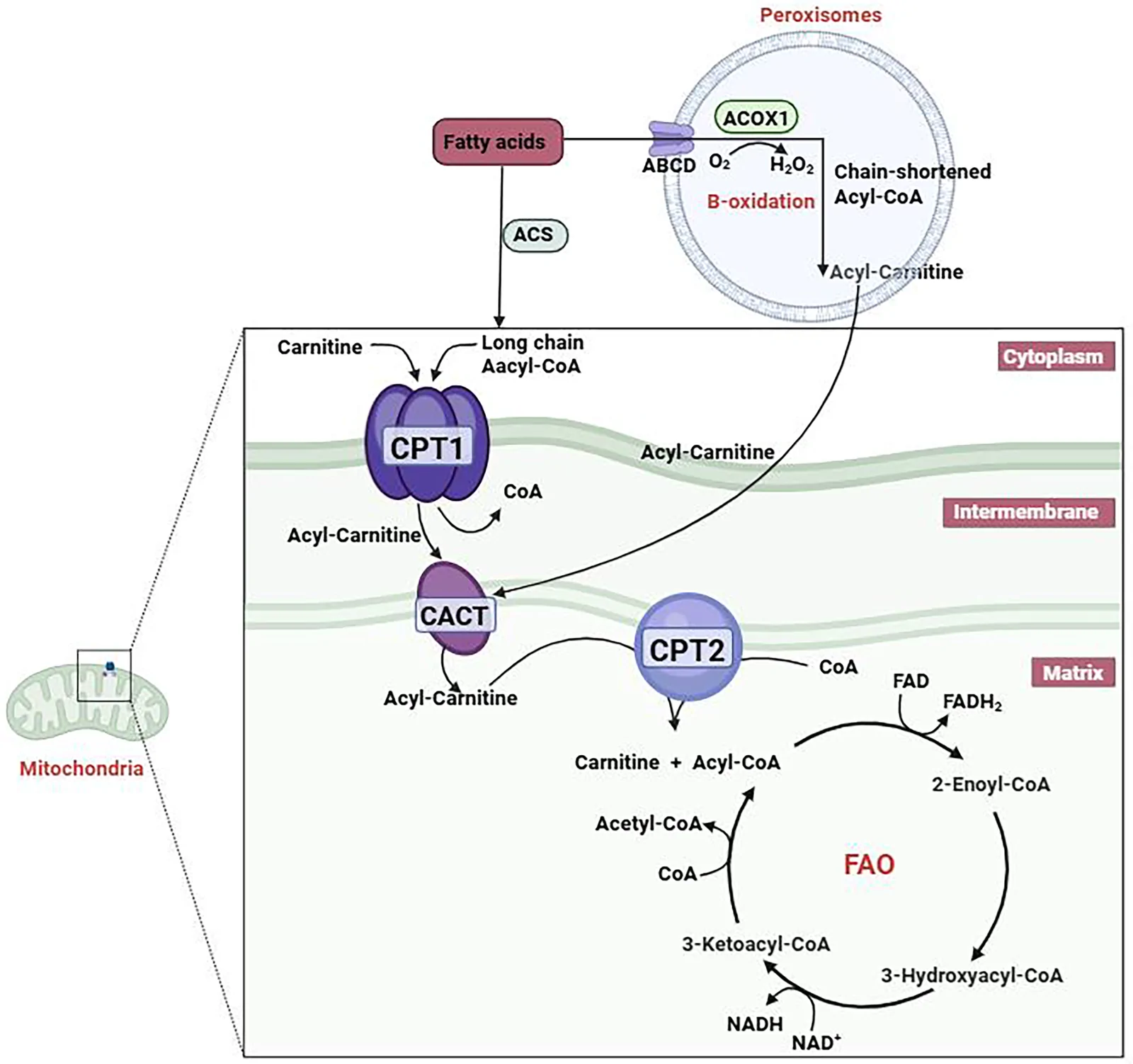
Mechanism of fatty acid oxidation. Cytosolic free fatty acids are activated by acyl-CoA synthetase (ACS) to long-chain acyl-CoA; very-long-chain fatty acids may first enter peroxisomes, where they are degraded via the β-oxidation pathway initiated by acyl-CoA oxidase 1 (ACOX1) into shorter-chain acyl-CoA species, which are converted to acylcarnitines and enter mitochondria for further metabolism. Long-chain acyl-CoA cannot freely cross the inner mitochondrial membrane and relies on the carnitine shuttle system for transmembrane transport: carnitine palmitoyltransferase 1 (CPT1), located on the outer mitochondrial membrane, catalyses the conjugation of acyl-CoA with carnitine to form acylcarnitine; acylcarnitine is transported into the mitochondrial matrix by carnitine–acylcarnitine translocase (CACT) on the inner membrane; and matrix-side carnitine palmitoyltransferase 2 (CPT2) catalyses the dissociation of acylcarnitine, regenerating acyl-CoA and free carnitine, which returns to the intermembrane space via CACT for recycling. The resulting acyl-CoA then enters the mitochondrial fatty acid β-oxidation (FAO) cycle for stepwise degradation, yielding acetyl-CoA that feeds the tricarboxylic acid cycle while generating the reducing equivalents FADH_2_ and NADH.

Under normal physiological conditions, the bulk of fatty acid β-oxidation in renal tissue is carried out by tubular epithelial cells, with a smaller fraction occurring in podocytes, whereas glomerular cells rely primarily on glucose metabolism and glycolysis to meet basal energy demands (19). CD36, a transmembrane protein of the class B scavenger receptor family, mediates fatty acid transport in adipocytes and myocytes and is highly expressed in tubular epithelial cells, podocytes and mesangial cells, where it indirectly governs mitochondrial β-oxidation homeostasis by regulating intracellular fatty acid trafficking (12, 20). CD36 cooperates with fatty acid transport proteins (FATPs) and fatty acid-binding proteins (FABPs) to transport fatty acids from the cytoplasm to mitochondria, where they undergo β-oxidation and the tricarboxylic acid cycle to supply energy for the kidney (21). In DKD, however, hyperglycaemia and insulin resistance disturb the dynamic balance between fatty acid synthesis and oxidation, precipitating lipid metabolic disorder (22). Renal lipid dysmetabolism causes excessive intracellular deposition of polyunsaturated fatty acids (PUFAs) and aggravates oxidative stress. The reaction between accumulated reactive oxygen species (ROS) and abnormally enriched PUFAs triggers a lipid peroxidation radical cascade that generates multiple highly toxic reactive aldehydes, producing lipotoxic alterations in the kidney (23). This process is intimately linked to the key pathological events of DKD, including disrupted mitochondrial quality control, mitochondrial metabolic reprogramming, ferroptosis, ER stress and gut microbiota perturbation. Together these processes orchestrate the onset and progression of renal disease, ultimately accelerating glomerulosclerosis, tubulointerstitial fibrosis and DKD progression.

## 3 Bidirectional regulation of mitochondrial quality control and fatty acid oxidation in diabetic kidney disease

Mitochondria are the principal site of fatty acid oxidation, and the mitochondrial quality control (MQC) system, the core machinery governing mitochondrial structural remodelling, metabolic operation and damage clearance, is essential for preserving mitochondrial function and intracellular homeostasis (24, 25). MQC generally encompasses mitochondrial biogenesis, mitochondrial dynamics and mitophagy, all of which participate in the regulation of FAO (26). Mitochondrial biogenesis supplies an adequate pool of functional mitochondria as the reaction platform for FAO; mitochondrial dynamics maintains normal mitochondrial morphology and the proper expression of FAO-related enzymes; and mitophagy removes damaged mitochondria, thereby preventing organelle accumulation and ROS bursts (27–29). Notably, the relationship between mitochondrial quality control and fatty acid oxidation is not unidirectional. Rather, they mutually amplify each other and jointly promote DKD progression (**Figure 2**) (30). However, their correlation in DKD still requires further experimental validation.

**Figure 2:**
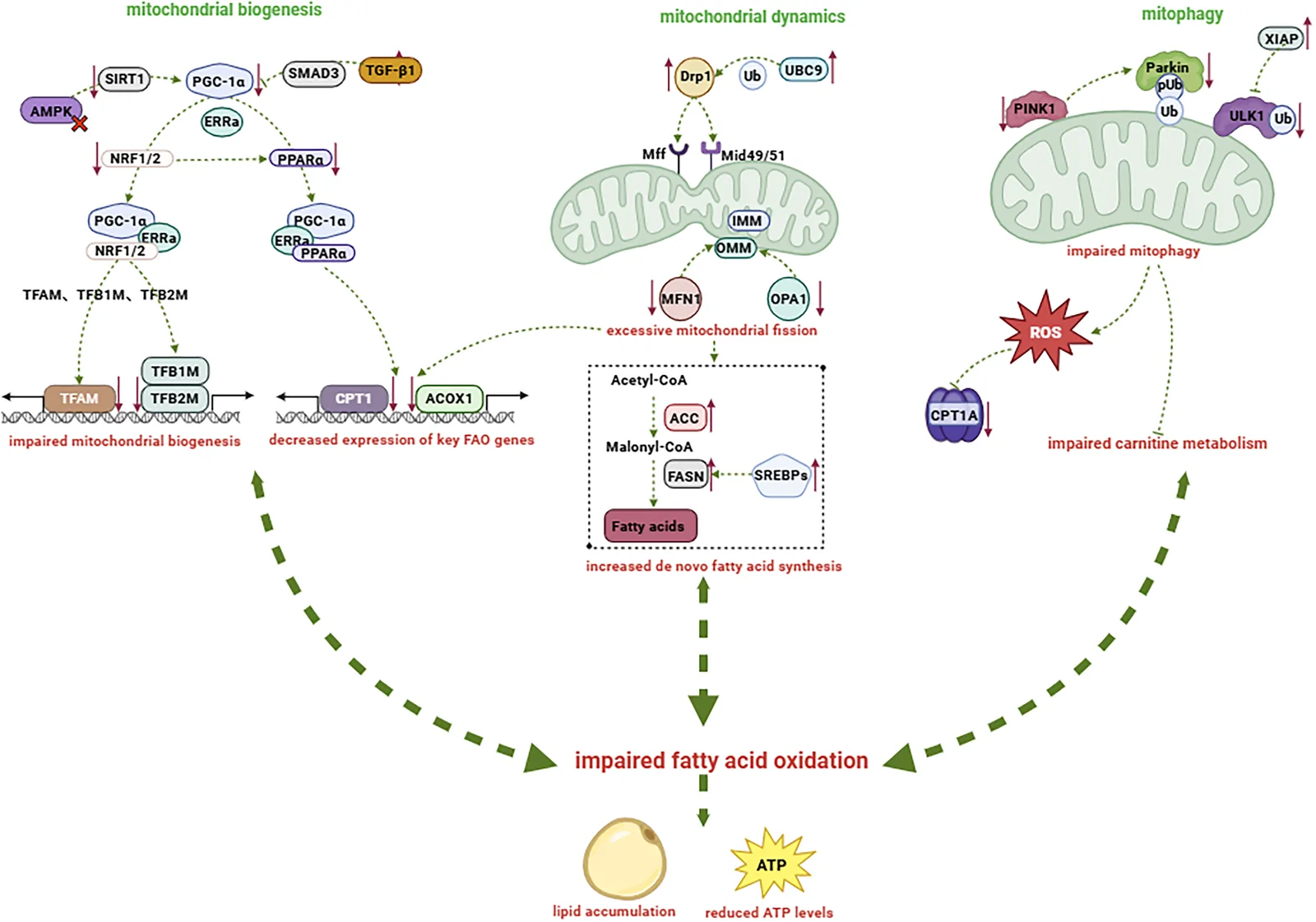
Disrupted mitochondrial quality control mediates fatty acid oxidation impairment in renal tubular epithelial cells. In the pathological state of diabetic kidney disease, the mitochondrial homeostasis regulatory network of renal tubular epithelial cells is perturbed across multiple dimensions, and three defects act in concert to drive FAO dysfunction. Impaired mitochondrial biogenesis: the AMPK/SIRT1 signalling pathway is suppressed, while activation of TGF-β1/SMAD3 signalling further inhibits the transcriptional regulatory functions of PGC-1α and its downstream effectors, leading to reduced mitochondrial neogenesis and downregulation of key FAO genes such as CPT1 and ACOX1. Imbalanced mitochondrial dynamics: expression and activity of the fission protein Drp1 rise while the fusion proteins Mfn1 and OPA1 decline, triggering excessive mitochondrial fission and network fragmentation; concomitant SREBP pathway activation upregulates ACC, FASN and other synthetic enzymes, increasing *de novo* fatty acid synthesis. Impaired mitophagy: dysfunction of PINK1/Parkin- and ULK1-mediated mitophagy prevents efficient clearance of damaged mitochondria and provokes reactive oxygen species (ROS) bursts; ROS inhibit CPT1A activity through oxidative modification and disturb carnitine metabolism. Together, these abnormalities produce mitochondrial FAO impairment, ultimately causing ectopic intracellular lipid accumulation and insufficient ATP generation, thereby driving tubular injury and the progression of renal fibrosis.

### 3.1 Mitochondrial biogenesis and fatty acid oxidation in DKD

Mitochondrial biogenesis refers to the generation of new mitochondria from pre-existing organelles through growth and division, a process that requires the synchronous synthesis of the inner and outer mitochondrial membranes, replication of the mitochondrial genome, and the synthesis and assembly of mitochondrial proteins (31). This intricate process is governed by multiple transcriptional cascade regulators, among which peroxisome proliferator-activated receptor-γ coactivator-1α (PGC-1α) is the most critical, participating in virtually every aspect of mitochondrial biogenesis (24). Upon activation by upstream pathways such as AMPK and SIRT1, PGC-1α stimulates the transcriptional activity of NRF1 and NRF2 in the nucleus, which in turn activate a series of key target genes including TFAM, TFB1M and TFB2M; mtDNA transcriptional activity and copy number increase concomitantly, providing the material basis for mitochondrial biogenesis (32, 33). Meanwhile, PPARα recruits PGC-1α to form a transcriptional complex, and together they synergistically activate FAO-related enzyme genes; inhibition of the SIRT1/PGC-1α/PPARα pathway significantly impairs FAO, suggesting that this pathway regulates FAO by modulating the synthesis of downstream key FAO enzymes (34, 35). Mitochondrial solute carrier family 25 member 35 (SLC25A35) promotes PGC-1α acetylation by increasing acyl-CoA levels, thereby enhancing PGC-1α expression and simultaneously augmenting FAO and mitochondrial biogenesis (36). These findings indicate that PGC-1α coordinates the two biological processes of FAO and mitochondrial biogenesis simultaneously.

In DKD, PGC-1α expression is downregulated in renal cells, its coactivator function is lost, PPARα transcriptional activity is reduced, and the expression of downstream key FAO genes decreases (37, 38). Inactivation of upstream AMPK and downregulation of SIRT1 have been observed in the DKD setting (39, 40); meanwhile, TGF-β1 is upregulated in DKD and directly represses PGC-1α via SMAD3, thereby suppressing mitochondrial biogenesis (41, 42). 3-Indolepropionic acid (IPA), derived from gut microbiota metabolism of dietary tryptophan, upregulates SIRT1 protein expression by blocking ubiquitin-mediated degradation; activated SIRT1 then activates PGC-1α-related signaling cascades, restoring mitochondrial FAO levels and promoting mitochondrial biogenesis in glomerular endothelial cells, thereby delaying DKD progression (43). Lipin-1 is a key regulatory protein maintaining lipid metabolic balance, and its deficiency causes renal injury and accelerates DKD. Mechanistically, Lipin-1 deficiency suppresses PGC-1α/PPARα-mediated CPT1α/hepatocyte nuclear factor 4α (HNF4α) signalling while markedly upregulating sterol regulatory element-binding proteins (SREBPs), thereby inhibiting FAO capacity and aggravating mitochondrial dysfunction, including biogenesis defects, to promote DKD progression (44). In DKD models, expression of the carnitine transporter OCTN2 is downregulated; the resulting carnitine deficiency impairs FAO and directly reduces PGC-1α expression, suppressing mitochondrial biogenesis and compromising respiratory chain function, ultimately culminating in renal fibrosis (45). In addition, NRF1 is downregulated in both patients with DKD and mouse models, impairing PPARα transcriptional activity, FAO and mitochondrial function (46). These findings suggest a close link between mitochondrial biogenesis and fatty acid oxidation in DKD, with PGC-1α acting as a key mediator connecting these two biological processes and representing a promising target for future amelioration of renal injury.

### 3.2 Mitochondrial dynamics and fatty acid oxidation in DKD

Mitochondrial dynamics determines the morphology, quality, quantity and distribution of mitochondria within the cell and comprises two opposing processes, fusion and fission (47). Mitochondrial fusion is the dynamic merging of two or more mitochondria into a single tubular organelle, mediated by three fusion factors: mitofusin 1 (MFN1), mitofusin 2 (MFN2) and optic atrophy 1 (OPA1) (48). These factors connect the inner (IMM) and outer (OMM) mitochondrial membranes to form membrane contact sites, coordinately regulating outer-membrane tethering and inner-membrane fusion (49). Mitochondrial fission is a quality-control process in which dynamin-related protein 1 (Drp1) is recruited to the outer mitochondrial membrane to form a constriction ring that severs the double membrane, yielding two daughter mitochondria (50). Cytosolic Drp1 translocates to the outer membrane in response to cellular signals, where it is recruited by Mff and Mid49/51 and oligomerizes into a helical ring structure that drives mitochondrial constriction and division through GTP hydrolysis (51, 52). Experiments combining respirometry with metabolic tracing have shown that Drp1 knockdown or Mfn overexpression alters mitochondrial morphology and thereby affects FAO (53). MFN2-mediated mitochondrial fusion enhances fatty acid transport into mitochondria, expands the mitochondrial matrix and promotes FAO; mechanistically, interaction between MFN2 and CPT1α through its GTPase domain, demonstrated using deletion mutants of both proteins, underlies this promotion of FAO (54). These observations indicate that mitochondrial dynamics factors can directly regulate the FAO process.

In renal tissue from patients with DKD and in experimental animal and cell models, MFN2 and OPA1 are downregulated whereas Drp1 is upregulated, leading to excessive mitochondrial fission, lipid accumulation and reduced cell viability (55, 56). Excessive mitochondrial fission promotes fatty acid synthesis by upregulating the lipogenic genes fatty acid synthase (FASN) and acetyl-CoA carboxylase 1 (ACC1) and by elongation of very-long-chain fatty acids protein 6 (ELOVL6), while simultaneously suppressing FAO through downregulation of CPT1A and ACOX1, thereby aggravating FAO impairment (57). Silencing Drp1 with small interfering RNA not only inhibited mitochondrial fragmentation but also reduced lipid accumulation and markedly restored FAO, an effect that was substantially reversed by a CPT1 inhibitor (58). Ubiquitin-conjugating enzyme E2I (UBC9), the only known SUMO E2-conjugating enzyme in mammals, has been identified as a core SUMOylation regulator of Drp1: SUMOylation of Drp1 by UBC9 intensifies mitochondrial fission and disrupts mitochondrial dynamics homeostasis. Conversely, targeted inhibition of UBC9 effectively modulates Drp1 SUMOylation, suppresses mitochondrial fission, restores FAO function, and is a key means of reducing ectopic lipid deposition in renal tubules and slowing DKD progression (59). Similarly, lipid metabolic disorders in DKD can disrupt mitochondrial dynamic homeostasis by altering mitochondrial membrane lipid composition and the expression of key regulatory proteins (60). Lipotoxicity induces excessive mitochondrial fragmentation, disrupts the fusion/fission balance, and leads to mitochondrial structural damage and dysfunction. The molecules involved include Drp1, Mfn2, ALCAT1, ChREBP, and others (30). In summary, modulating fatty acid oxidation capacity through the maintenance of mitochondrial dynamic homeostasis has emerged as a promising therapeutic strategy, offering a new direction for future research into effective approaches to slow DKD progression.

### 3.3 Mitophagy and fatty acid oxidation in DKD

Mitophagy is a subtype of selective autophagy that relies on the cellular autophagy machinery to specifically engulf and eliminate structurally damaged or depolarized mitochondria, thereby preserving intracellular homeostasis (61). This process is vital for maintaining oxidative phosphorylation (OXPHOS), ATP synthesis, calcium regulation, ROS generation and mitochondrial DNA maintenance (62). According to its molecular initiation mechanism, mitophagy is generally divided into ubiquitin-dependent and ubiquitin-independent pathways, of which the PINK1/Parkin pathway is the most extensively studied ubiquitin-dependent route (62). In DKD, lipid metabolic disorders can disrupt the structure and function of mitochondrial membranes, thereby activating mitophagy to remove damaged mitochondria (30). Therefore, maintaining the functional integrity of mitophagy is particularly critical for renal cell survival.

In DKD, PINK1/Parkin-mediated mitophagy is markedly suppressed. In DKD mouse models and in HK-2 cells treated with high glucose/high fatty acids, mitochondrial expression of Pink1, Parkin, Atg5 and LC3II proteins is significantly downregulated (63). As mitophagy becomes dysfunctional, damaged mitochondria cannot be cleared efficiently, resulting in ROS bursts (64), which in turn are a major trigger for the inactivation of key FAO enzymes such as CPT1A (65). Beyond removing damaged organelles, mitophagy also directly influences DKD progression by regulating lipid metabolism. Analyses of renal biopsies from patients with DKD, diabetic mouse models and high-glucose-treated tubular epithelial cells have revealed that XIAP–ULK1-mediated mitophagy alleviates DKD by modulating carnitine metabolism (66). Carnitine is the key carrier for the entry of long-chain fatty acids into mitochondria for β-oxidation, and its metabolic homeostasis is tightly coupled to mitophagy. When mitophagy is compromised, disturbed carnitine metabolism further exacerbates FAO impairment and lipid accumulation (45). In DKD model mice, expression of the lipid droplet membrane protein perilipin-2 and lipid deposition are markedly elevated, whereas SIRT1 expression is significantly reduced and negatively correlated with lipid deposition (67). SIRT1 overexpression promotes mitophagy and attenuates lipotoxicity-induced ferroptosis in renal tubular epithelial cells (67), further confirming the therapeutic potential of enhancing mitophagy to ameliorate lipid metabolic disorder in DKD.

Unlike proximal tubular epithelial cells, podocytes and mesangial cells are less dependent on FAO, yet their autophagic regulation also affects DKD progression. Podocytes exhibit relatively high basal autophagy levels, and autophagic abnormalities are closely associated with podocyte injury and proteinuria. Mesangial cell autophagy can negatively regulate extracellular matrix deposition and inhibit glomerulosclerosis, whereas in proximal tubular cells under diabetic conditions, autophagic dysfunction can lead to mitochondrial damage and interstitial fibrosis (68). In a podocyte injury model, it was further confirmed that high glucose combined with hydrogen peroxide stimulation reduced the LC3II/LC3I ratio and p62 expression in podocytes, indicating impaired autophagic flux (69). This study provides direct cellular experimental evidence for the regulation of autophagic flux in podocytes and suggests that the mechanisms regulating autophagy in podocytes may differ from those in tubular cells. Furthermore, a single-cell transcriptomic analysis revealed the heterogeneity of immune cells and tubular epithelial cells and their intercellular communication network in DKD kidneys, suggesting that metabolic and autophagic states may differ significantly among cell types in the DKD microenvironment (70). In summary, autophagy regulation is cell-type specific, and future studies are needed to further dissect the interplay between mitophagy and FAO in specific cell types.

## 4 Interplay between defective fatty acid oxidation and renal metabolic reprogramming in DKD

Metabolic reprogramming refers to the capacity of cells to adaptively remodel their metabolic processes in response to environmental change (71). As the central hub of cellular metabolism, mitochondria are the principal executors of this reprogramming, and shifts in their substrate utilization preference, spanning lipid, glucose and amino acid metabolic pathways, are particularly consequential (72). These three nutrient pathways act in concert during the development and progression of DKD to maintain mitochondrial metabolic homeostasis. Current evidence indicates that the regulation of mitochondrial function by FAO extends beyond the MQC system and also mediates the metabolic remodelling of renal cells within the mitochondrial metabolic reprogramming of DKD (73). Defective fatty acid oxidation and renal metabolic reprogramming exhibit bidirectional crosstalk. Impaired fatty acid oxidation creates an energy deficit that drives compensatory glycolysis, lactate accumulation, and impaired branched-chain amino acid catabolism; conversely, metabolic reprogramming suppresses fatty acid oxidation. It is worth noting that this metabolic interplay is not an isolated event within a single cell. Single-cell transcriptomic studies have revealed marked immune cell heterogeneity in the DKD renal microenvironment, where tubular epithelial cells, macrophages, T cells, and other cells form a multidirectional interaction network through signals such as GRN/SORT1 and CD74 (70). This suggests that the interplay between defective fatty acid oxidation and metabolic reprogramming may be embedded within a communication network composed of multiple renal and immune cells, thereby participating in DKD microenvironment remodeling.

### 4.1 Lipid metabolic reprogramming and fatty acid oxidation in DKD: FAO impairment and aberrant lipid droplet accumulation

Lipid metabolic reprogramming denotes the wholesale remodelling of lipid uptake, synthesis, storage and catabolism in renal cells within the diabetic microenvironment. The kidney is one of the most energetically active organs, and tubular epithelial cells rely on mitochondrial FAO as their main source of ATP (18). FAO is a pivotal component of lipid metabolism; once it is impaired and free fatty acids cannot be properly catabolized, lipid droplets (LDs) accumulate abnormally, precipitating lipid metabolic reprogramming (74). In DKD, the kidney shifts from a fatty-acid-oxidizing organ to a lipid-storing organ, a transition that constitutes the core feature of lipid metabolic reprogramming in this disease (74, 75).

Renal cells undergo pronounced lipid metabolic reprogramming in the DKD microenvironment, characterized by increased lipid uptake driven by upregulation of CD36 and FATP (76, 77). Kidney injury molecule-1 (KIM-1) has recently been identified as a novel fatty acid uptake receptor that is highly expressed in the proximal tubules of DKD and mediates the endocytic entry of free fatty acids into cells, directly promoting lipid accumulation and disease progression (78). In addition, SREBP1/2-driven *de novo* lipogenesis is activated, with elevated ACC and FASN expression further increasing the intracellular lipid burden (79). The increased lipid supply should in principle be matched by a compensatory rise in FAO; in DKD, however, FAO is systemically suppressed, creating a metabolic bottleneck of “oversupply coupled with blocked utilization”. Downregulation of PGC-1α and CPT1A in DKD models directly determines the metabolic fate of fatty acids, diverting them from oxidative degradation towards esterification and storage (44, 80). The immediate consequences of FAO suppression are twofold. On the one hand, tubular epithelial cells are deprived of their preferred ATP source and an energy crisis ensues; on the other, unoxidized fatty acids are shunted into esterification pathways, promoting lipid droplet formation and the generation of lipotoxic intermediates (65). Genetic deletion of CD36 has been shown to restore FAO and improve mitochondrial function, further confirming the causal coupling between increased uptake and impaired oxidation (76). When lipid overload persists without a matching increase in oxidative and efflux capacity, chronic LD accumulation becomes a pathological signal. Lipidomic analyses in DKD indicate that the accumulated lipids are predominantly triglycerides and correlate negatively with renal function (81, 82). Persistent LDs are closely associated with oxidative stress, dysregulation of perilipin family proteins and cellular injury (83). Through a lipotoxicity–inflammation–quality-control-collapse–programmed-cell-death cascade, pathological LD accumulation in the DKD kidney converts FAO impairment into a key intermediate hub of structural renal damage (12). Under high-glucose conditions, oxidative stress in HK-2 cells intensifies; the activity of factors involved in sphingosine-1-phosphate (S1P) synthesis (S1pr1, SPHK1, Acer1 and others) rises markedly, whereas Spns2 expression is suppressed, leading to S1P deposition, lipid droplet generation, enhanced lipid peroxidation and impaired FAO. These changes drive lipid metabolic reprogramming and accelerate DKD, and SS31, a tetrapeptide that protects mitochondrial cristae, reverses them by inhibiting S1P-synthetic factor activity and reducing S1P deposition (84). FKBP51, a key regulator of lipid metabolic balance, also participates in lipid metabolic reprogramming: FKBP51 knockdown aggravates intracellular lipid accumulation, whereas its overexpression reverses palmitate-induced lipid deposition (85). Furthermore, maresin 1 (MaR1), a specialized pro-resolving lipid mediator that is downregulated in patients with DKD, normally alleviates inflammation in DKD models via the LGR6-mediated cAMP–SOD2 antioxidant pathway (86), suggesting that MaR1 may become a potential therapeutic target for DKD. Collectively, these studies demonstrate that FAO can be harnessed to intervene in DKD by modulating lipid metabolism.

### 4.2 Glucose metabolic reprogramming and fatty acid oxidation in DKD: compensatory enhancement of glycolysis driven by oxidative impairment

Glucose metabolism encompasses glycolysis, the TCA cycle and the pentose phosphate pathway (PPP), and constitutes the second most efficient energy-supply route after FAO, serving as a core compensatory pathway that maintains cellular energy homeostasis when lipid metabolism is compromised (87). Glucose is phosphorylated by hexokinase to glucose-6-phosphate (G6P), which can either proceed through glycolysis to pyruvate or be diverted into the PPP to generate ribose-5-phosphate (R5P) for nucleotide synthesis and NADPH for redox balance. Under aerobic conditions pyruvate mainly enters the mitochondrial TCA cycle after conversion to acetyl-CoA, whereas under hypoxia it is reduced to lactate (72, 88, 89). Perturbation of cellular microenvironmental homeostasis can unbalance these intrinsic glucose metabolic routes, manifesting as the synchronous abnormal activation of glycolysis and gluconeogenesis with substantial intracellular lactate accumulation, ultimately driving glucose metabolic reprogramming (90). A pharmacological study found that blocking FAO activates the mTORC1 pathway through GCN5-mediated acetylation of Raptor, markedly enhancing glycolysis and substantially raising intracellular acetyl-CoA content (73). Suppressed FAO therefore directly drives glucose metabolic reprogramming, with cells increasing glucose catabolism to compensate for the energy deficit, and aberrant glucose metabolic reprogramming likewise occurs in DKD (21).

In streptozotocin (STZ)-induced diabetic mice, the transcription and protein levels of FAO-related genes, including PPARα, CPT1α and ACADL, are markedly downregulated, whereas the expression of glycolysis-related enzymes such as HK2, LDH and PDK1 is significantly increased, accompanied by a pronounced rise in renal lactate content (91). HIF-1α plays a central role in this metabolic switch. Its activation contributes to the expression of glycolytic target genes (GLUT1, HK2, PFKFB3, LDHA, and PKM2) and lactate transport. In addition, HIF-1α can directly bind to the regulatory region of the PGC-1α promoter, suppressing PGC-1α expression and establishing a feed-forward mechanism whereby glycolytic reprogramming further reinforces the inhibition of fatty acid oxidation (65, 92). siRNA-mediated knockdown of HIF-1α substantially reverses high-glucose-induced lipid droplet accumulation, restores the expression of Ppara and its downstream FAO enzymes, suppresses the rise of glycolytic enzymes and reduces lactate production (91). In proximal tubular epithelial cells, expression of the deacylase SIRT5 increases; SIRT5 removes malonylation from glycolytic enzymes, enhancing their activity and promoting glycolysis (93), which may explain the metabolic switch in DKD. Increased glycolysis generates various metabolites and profibrotic factors that promote tubular fibrosis (94). Likewise, the accumulation of lactate, a glycolytic by-product, can trigger lactylation of fission protein 1 (Fis1), leading to increased mitochondrial fission, excessive mitochondrial ROS production and mitochondrial dysregulation. Consistent with this, mitochondrial proteins in the kidneys of db/db mice and patients with DKD broadly harbour lysine lactylation; in high-glucose-cultured HK-2 cells, aberrant upregulation of ACSF2 drives compensatory glycolytic hyperactivation by blocking mitochondrial FAO, and the resulting massive lactate accumulation further intensifies lysine lactylation, advancing DKD (95, 96). Notably, in mice with tubule-specific adiponectin overexpression, FAO pathway activity is markedly upregulated and pyruvate-dependent gluconeogenesis is enhanced, an enhancement that is abolished when FAO is inhibited, demonstrating that FAO exerts important regulatory control over renal gluconeogenesis (97).

### 4.3 Amino acid metabolic reprogramming and fatty acid oxidation in DKD: branched-chain amino acid accumulation and oxidative suppression

Amino acids are organic compounds bearing both amino and carboxyl functional groups. As the material basis of protein synthesis, their metabolic homeostasis directly governs numerous physiological functions, including biosynthesis and neural signalling (98). The oxidative catabolism of amino acids begins with aminotransferase-mediated deamination: the α-amino group is transferred to α-ketoglutarate to yield glutamate, which then enters the urea cycle for amino-group disposal and urea production; the remaining carbon skeletons feed into the citric acid cycle as keto acids and are fully oxidized to carbon dioxide with energy release. This process is not only an important supplementary route for cellular ATP acquisition but also a core metabolic process sustaining normal cell growth, differentiation and organismal homeostasis (98, 99). Pathological stimuli can disrupt intracellular homeostasis and remodel the uptake, transport, catabolism and anabolism of amino acids as a whole, inducing amino acid metabolic reprogramming (100). In mice lacking long-chain acyl-CoA dehydrogenase (LCAD KO), fasting not only suppresses gluconeogenesis but also weakens branched-chain amino acid metabolism, demonstrating that FAO markedly influences amino acid metabolism (101). Emerging evidence indicates that the kidney plays a key role throughout amino acid metabolism, including synthesis, catabolism, filtration and reabsorption, particularly in metabolic diseases such as DKD (102).

Branched-chain amino acids (BCAAs: leucine, isoleucine and valine) are essential amino acids that account for roughly 15–25% of dietary protein intake (103). In DKD, BCAA catabolism is impaired and plasma BCAA levels rise significantly; BCAAs and their keto-acid derivatives are being investigated as potential predictive biomarkers of DKD (104, 105). Branched-chain ketoacid dehydrogenase kinase (BCKDK) is a key negative regulator of BCAA catabolism. In DKD, aberrantly high BCKDK expression markedly suppresses leucine degradation, leading to substantial leucine accumulation in renal tubules. The accumulated leucine induces tubular metabolic reprogramming via activation of the mTOR signaling pathway, inhibits fatty acid oxidation, and forms a positive feedback loop that exacerbates aberrant lipid accumulation, thereby mediating tubular injury and DKD progression. This suggests that amino acid metabolic reprogramming participates in DKD pathogenesis by regulating lipid metabolic homeostasis (106). Tryptophan is metabolized mainly through the kynurenine pathway, whose activation in DKD is closely associated with inflammation, endothelial dysfunction and fibrosis; accumulation of kynurenine pathway metabolites promotes inflammation and oxidative stress, aggravating kidney injury (107). Gut microbial tryptophan metabolites, such as the uraemic toxin indoxyl sulfate, accumulate markedly as renal function declines and exacerbate tubular injury by inducing oxidative stress and promoting fibrosis (108). Serine appears relatively deficient in DKD, which may compromise one-carbon metabolism and antioxidant defence (105); serine deficiency weakens the capacity of cells to scavenge ROS by impairing glutathione (GSH) synthesis, indirectly aggravating the oxidative damage accompanying FAO impairment (109). ERRα, a master transcriptional regulator of FAO, is specifically downregulated in patients with DKD and in mouse models (110). Specific knockout of ERRα markedly reduces NOS expression in HK-2 cells and lowers intracellular citrulline content, further aggravating cellular injury and accelerating senescence, whereas ERRα overexpression remodels citrulline metabolic homeostasis and reverses cellular injury (111). In addition, dietary interventions targeting amino acids can correct metabolic disturbances in DKD and upregulate fatty acid metabolism. Methionine-restricted diets restore the expression of the colonic barrier proteins Claudin-1, ZO-1 and MUC2 in DKD models, repairing intestinal barrier integrity; they also remodel gut microbiota composition, enriching short-chain-fatty-acid-producing bacteria and increasing propionate production, which activates FAO through the propionate/GPR41 signalling pathway and improves renal function (112).

## 5 Distal pathological interactions and systemic regulation mediated by impaired fatty acid oxidation

### 5.1 Mechanisms linking defective fatty acid oxidation to ferroptosis in DKD

Ferroptosis is a non-apoptotic form of programmed cell death that constitutes a core pathological mechanism underlying tubular injury and glomerular lesions in DKD; its essence lies in the massive accumulation of membrane lipid peroxides catalysed by iron ions (113, 114). The process can be divided into a sensitivity-regulation phase and a death-execution phase. The execution phase centres on membrane lipid peroxidation: phospholipid hydroperoxides are generated in abundance via lipoxygenases or radical-mediated non-enzymatic routes, then interfere with calcium and sodium transport mediated by Piezo and TRP channels and inhibit Na^+^/K^+^-ATPase activity, provoking osmotic imbalance and cell swelling; increased membrane rigidity further activates ion channels through positive feedback until membrane rupture occurs (115). Sensitivity regulation depends chiefly on two antioxidant pathways. First, the cystine/glutamate antiporter (system xc^-^) imports cystine for GSH synthesis, and GPX4 uses GSH to reduce toxic phospholipid hydroperoxides to harmless lipid alcohols. Second, ferroptosis suppressor protein 1 (FSP1) relies on NAD(P)H to reduce antioxidant molecules such as coenzyme Q10 and vitamin K, directly neutralizing phospholipid peroxyl radicals and interrupting the lipid peroxidation chain reaction (116, 117). An intrinsic pathological connection exists between FAO deficiency and ferroptosis. FAO is the main pathway by which tubular epithelial cells maintain energy homeostasis, whereas lipid peroxidation, the core driver of ferroptosis, depends precisely on the content and distribution of PUFAs within specific membrane phospholipids (41, 118). ACSL4 and LPCAT3 are the key enzymes incorporating PUFAs into membrane phospholipids, and their activity directly determines cellular sensitivity to ferroptosis (119). In DKD, FAO suppression causes abnormal intracellular lipid accumulation, and these lipids, particularly PUFA-rich phospholipids, provide ample substrate for lipid peroxidation (120). Meanwhile, mitochondrial dysfunction caused by FAO impairment further intensifies ROS generation, which is a prerequisite for initiating the lipid peroxidation chain reaction (22). FAO deficiency therefore promotes ferroptosis through two convergent routes: increased substrate supply and amplified oxidative pressure.

Ferroptosis has been confirmed to play a key role in the onset and treatment of various kidney diseases and to participate in regulating the course of DKD (121). In the kidneys of STZ-induced type 1 diabetic mice and db/db mice, ferroptosis markers are markedly elevated, and the ACSL4 inhibitor rosiglitazone improves renal function while reducing lipid peroxidation products and iron content (122). Further work has shown that SLC7A11 and GPX4 expression is significantly downregulated in renal biopsies from patients with DKD, whereas iron content and toxic lipid peroxide levels are elevated in TGF-β1-stimulated NRK-52E cells and in the kidneys of type 1 diabetic mice (123). Fatty acid-binding protein 4 (FABP4) is highly expressed in DKD renal tissue; by inhibiting CPT1A- and PPARγ-mediated FAO, it causes lipid deposition and lipid peroxidation, ultimately inducing ferroptosis in HK2 cells (120). STAT1 suppresses FAO while concurrently downregulating the ferroptosis inhibitors SLC3A2 and SLC7A11, thereby promoting ferroptosis and kidney injury on two fronts (124). Augmenter of liver regeneration (ALR) is a secreted protein localized to the inner mitochondrial membrane and is widely involved in regulating pathological injury in kidney diseases. ALR can directly bind to CPT1A and promote its expression. When ALR expression is deficient, CPT1A expression is reduced and FAO is suppressed, thereby promoting ferroptosis and macrophage activation and accelerating DKD progression (125, 126). Conversely, normal ALR expression suppresses ferroptosis and exerts renoprotective effects (125). In addition, calcium/calmodulin-dependent protein kinase kinase 2 (CaMKK2), a serine/threonine kinase upstream of calcium signaling, promotes lipid peroxidation and causes mitochondrial damage in DKD. β-hydroxybutyrate (BHB) can reverse this damage by inhibiting CaMKK2 expression, significantly ameliorating fatty acid peroxidation and mitochondrial dysfunction. These findings suggest that CaMKK2 is a potential target for regulating ferroptosis in DKD (127). Notably, studies on organ fibrosis have indicated that the role of ferroptosis in fibrosis is cell-type-dependent and bidirectionally regulated (128). This recognition suggests that when evaluating the FAO–ferroptosis axis in future DKD studies, the specific metabolic contexts of tubular epithelial cells, macrophages, and other renal cells should be distinguished.

This apparent paradox — that both impaired and enhanced FAO can aggravate ferroptosis — is resolved when three variables are considered. First, substrate identity and PUFA supply: β-oxidation of saturated acyl-CoAs yields acetyl-CoA and reducing equivalents without generating oxidizable PUFAs, whereas mobilization of PUFA-containing phospholipids provides substrate for ACSL4- and LPCAT3-mediated esterification, so that the size of the peroxidizable lipid pool is set by the balance between FAO flux and PUFA availability rather than by FAO activity alone. Second, electron transport status and ROS load: when the respiratory chain and the GPX4/GSH and FSP1/CoQ10 systems are competent, a higher FAO flux is not pro-ferroptotic; when electron leak at complexes I/II coexists with antioxidant insufficiency, as occurs in DKD, accelerated FAO increases mitochondrial ROS and labile iron and tips the balance towards lipid peroxidation. Third, cell type and disease stage: proximal tubular epithelial cells are highly FAO-dependent whereas podocytes, glomerular endothelial cells and mesangial cells are not, so an identical change in FAO flux produces different effects on the labile-iron/PUFA/antioxidant balance across cell types, and the consequences also differ between the sensitivity-regulation and death-execution phases of ferroptosis. Accordingly, FAO deficiency (substrate accumulation) and FAO enhancement (oxidative flux) can both aggravate ferroptosis, depending on whether the rate-limiting constraint lies in substrate supply or in the oxidative–antioxidant balance. Distinguishing these contexts will be essential for interpreting the FAO–ferroptosis axis and for designing cell-type-specific interventions.

### 5.2 Interplay between defective fatty acid oxidation and endoplasmic reticulum stress in DKD

The endoplasmic reticulum (ER) is the core organelle governing protein synthesis, folding, trafficking and post-translational modification, and is essential for the orderly conduct of cellular physiology (129). Endogenous or exogenous stimuli can impair ER protein folding, disrupt ER homeostasis and cause the accumulation of large quantities of misfolded or unfolded proteins, a pathophysiological alteration termed ER stress (130). Persistent ER stress is an important contributor to the development of multiple kidney diseases and progressively advances renal fibrosis (129). Studies reveal a bidirectional regulatory relationship between ER stress and lipid metabolism: the unfolded protein response (UPR) not only governs protein quality control but also preserves energy balance and membrane integrity by reprogramming lipid synthesis, oxidation and storage; conversely, disturbed lipid metabolism can disrupt ER homeostasis and amplify stress signalling, forming a feedback loop of metabolic and proteostatic imbalance (131).

In DKD, FAO deficiency and ER stress form a mutually reinforcing vicious cycle. On the one hand, FAO suppression causes intracellular accumulation of free fatty acids, among which saturated fatty acids such as palmitate can be incorporated into the ER membrane, increasing membrane rigidity, reducing fluidity, and depleting ER calcium by inhibiting SERCA pump activity or promoting calcium efflux; molecular chaperones are thereby inactivated and the accumulation of misfolded proteins intensifies (45, 131). In proximal tubular epithelial cells, ER stress elicited by saturated fatty acid loading has been established as a core mechanism of lipotoxic injury. Animal experiments further confirm that palmitate treatment markedly raises C16:0 ceramide levels in tubular cells, directly triggering ER stress through altered ER membrane fluidity (132). On the other hand, multilayered bidirectional interactions exist between the three UPR signalling branches and FAO regulation. The PERK–eIF2α–ATF4 axis relieves the repression of SREBP1 by inhibiting Insig-1 translation, promoting the expression of key lipogenic enzymes including FASN, ACC and stearoyl-CoA desaturase 1 (SCD1) (131); it can also induce CHOP, which promotes apoptosis by repressing anti-apoptotic Bcl-2 family proteins and increasing oxidative stress (133). In the DKD setting, sustained activation of this pathway not only intensifies *de novo* lipogenesis but also aggravates lipid peroxidation. The IRE1α–XBP1 axis directly induces the expression of lipogenic enzymes such as ACC via XBP1s, promoting fatty acid synthesis and membrane expansion (134); at the same time, IRE1α selectively degrades mRNAs such as DGAT2 through regulated IRE1α-dependent decay (RIDD), modulating lipid droplet formation and lipid storage (135). In DKD, persistent IRE1α activation instead promotes inflammation and apoptosis through JNK and NF-κB signalling (133). Although the ATF6 pathway mainly mediates a protective ER response, aberrant ATF6α activation downregulates PPARα expression, impairs fatty acid metabolism in proximal tubular cells, and thereby promotes lipotoxicity-induced apoptosis and tubulointerstitial fibrosis (136).

The structural integrity of ER–mitochondria contact sites (mitochondria-associated membranes, MAMs) plays a key role in the interplay between FAO and ER stress. In DKD, structural and functional abnormalities of ER–mitochondria contacts are closely related to UPR dysregulation (137). Disruption of MAM structure, on the one hand, interferes with fatty acid transfer and oxidation between lipid droplets and mitochondria (138); on the other hand, it aggravates ER stress by perturbing ER–mitochondria calcium signalling (139). This dual structural and functional disturbance perpetuates and amplifies the vicious cycle between FAO deficiency and ER stress. Of note, dipeptidyl peptidase-4 (DPP-4) is a serine protease, and the SREBP-1/CD36 pathway is upregulated in DKD rats, promoting fatty acid uptake by renal tubules and podocytes, aggravating ectopic lipid deposition, and inducing ER stress and related renal lesions that drive DKD progression. Inhibiting DPP-4 expression markedly reverses these pathological changes (140, 141).

### 5.3 Regulation by the gut microbiota in the context of defective fatty acid oxidation in DKD

Recent studies have revealed a complex interaction network between the gut microbiota and its derived metabolites on the one hand and metabolic organs such as the liver and kidney on the other, through which the microbiota can participate in the initiation and progression of metabolic diseases including DKD (142, 143). The gut microbiota is a key component in maintaining organismal homeostasis and broadly regulates inflammation, lipid metabolism and oxidative stress (144–146). Extensive research on the gut–kidney axis has provided a deeper interpretation of the symbiotic relationship between the gut microbiota and the kidney (147). Short-chain fatty acids (SCFAs), metabolites generated by anaerobic fermentation of the gut microbiota, positively regulate FAO and ameliorate lipid metabolic disorder (148). In DKD, structural imbalance of the gut microbiota can trigger a persistent inflammatory cascade, while insufficient SCFA synthesis directly impairs epigenetic regulatory functions, further mediating renal fibrosis and cellular senescence (149).

Patients with DKD exhibit marked gut microbiota dysbiosis, with characteristic changes in microbial diversity and composition compared with patients who have diabetes alone (150). Dysbiosis is accompanied by significantly reduced SCFA production. Clinical studies confirm that faecal and serum SCFA levels in patients with DKD are significantly lower than in diabetic patients without nephropathy and in healthy controls, and that SCFA levels correlate negatively with renal function (151), suggesting that dysbiosis is not merely a concomitant phenomenon but may be an active contributor to disease progression. One team found that exogenous SCFAs, butyrate in particular, suppress oxidative stress and NF-κB inflammatory signalling through GPR43 (G protein-coupled receptor 43)-mediated pathways, improving albuminuria, serum creatinine and blood urea nitrogen in diabetic mice and inhibiting mesangial matrix accumulation and renal fibrosis (152). Dietary fibre supplementation delays DKD progression by promoting the proliferation of SCFA-producing bacteria, raising faecal and serum SCFA levels, and activating the GPR43 and GPR109A receptors (153). In STZ-induced diabetic rats, butyrate improves renal pathology and oxidative stress (153). A direct consequence of gut dysbiosis is impairment of the intestinal barrier. Meanwhile, inefficient proximal tubular FAO has been positioned as the primary cause of diabetic tubular injury and fibrosis (154). As renal function deteriorates, accumulating uraemic toxins likewise affect intestinal barrier function (155). In DKD, intestinal tight junction proteins such as ZO-1 and occludin are downregulated, compromising the integrity of the intestinal mucosal barrier and producing a so-called “leaky gut” state (156). This alteration allows lipopolysaccharide (LPS), a cell-wall component of Gram-negative bacteria, to cross the intestinal barrier into the circulation, where it activates Toll-like receptor 4 (TLR4) and its downstream NF-κB signalling, inducing systemic low-grade inflammation (156). Notably, activation of the TLR4/NF-κB pathway has been shown to directly inhibit the renal PPARα/PGC-1α/CPT1A signalling axis, thereby weakening FAO (157). Gut dysbiosis additionally affects bile acid metabolism. Secondary bile acids are key metabolites produced when gut microbes 7α-dehydroxylate primary bile acids. In patients with DKD, the abundance of bacteria involved in bile acid transformation, such as 7α-dehydroxylase-producing species, is altered, resulting in an abnormal bile acid profile (143). As endogenous ligands of FXR and TGR5, disturbed bile acids can directly or indirectly affect renal lipid metabolism and mitochondrial function. Activation of FXR/TGR5 signalling has been shown to promote renal FAO and improve lipid deposition, whereas impaired bile acid signalling in DKD may further aggravate FAO impairment (158).

## 6 Therapeutic targeting of fatty acid oxidation in DKD

Current research on targeting FAO in DKD spans natural compounds, modern pharmaceuticals, Chinese patent medicines and lifestyle interventions. These modalities act jointly to regulate FAO homeostasis, ameliorate disease-associated pathological processes and delay DKD progression (**Table 1**).

**Table 1:** Representative interventions targeting fatty acid oxidation in DKD and their mechanisms.

Category	Intervention	Mode of action	Target	Mechanism of action	Evidence type	Species/model limitations	Reference
Natural compounds	Stachyose	Indirect	SGLT2 (Tyr270 site)	Induces SGLT2 degradation, reverses pathological metabolic reprogramming, and restores FAO	Causal	Cell + Rodent; human-mouse conservation of SGLT2 site and human effects remain to be validated	(175)
	Quercetin	Direct	PPARA/PPARG-UCP1	Upregulates FAO, reduces ROS accumulation, and alleviates lipid deposition and fibrosis	Causal	Cell + Rodent; human validation is needed.	(161)
	Berberine	Direct	PGC-1α	Promotes FAO, inhibits lipid deposition and mitochondrial ROS, and restores mitochondrial energy homeostasis	Causal	Cell + Rodent; human validation is needed	(163, 164)
	Boeravinone C	Direct	PPARα/CPT1A/ACOX1	Reverses PPARα inhibition, upregulates key enzymes of FAO, and inhibits renal tubular apoptosis	Causal	Cell + Rodent; human validation is needed	(75)
	Eicosapentaenoic acid	Indirect	PKCβ/TGF-β/PAI-1	Regulates CD31 expression, inhibits EndMT, and maintains fatty acid homeostasis	Causal	Cell + Rodent; direct FAO evidence limited	(176)
	Ergosterol	Direct	CPT1A	Ameliorates FAO disorder and alleviates renal tissue injury	Causal	Cell + Rodent; human validation needed	(165)
	Fucoidan	Indirect	AhR/CD36	Modulates oxidative stress and amino acid metabolism; its effect on FAO remains to be verified	Correlative	Rodent;FAO direction in original needs verification	(177)
	Curcumin/piperine composite	Indirect	Nrf2/HO-1	Elevates butyrate levels, suppresses inflammation and oxidative stress, and preserves intestinal-renal barrier	Correlative	Cell + Rodent; causal chain unclear	(178)
Modern drugs	SGLT2 inhibitors (canagliflozin, etc.)	Indirect	AMPK/NRF2/GPX4; HIF-1α	Inhibits ferroptosis, upregulates FAO, and reverses the metabolic shift from FAO to glycolysis	Causal	Human plasma + Cell + Rodent; clinical hard endpoints need validation	(91, 179, 180)
	GLP-1 receptor agonists (semaglutide)	Indirect	cAMP/PKA/CREB→AMPK	Upregulates KLB expression, remodels lipid metabolism, and inhibits ferroptosis, inflammation and fibrosis	Causal	Human plasma + Cell + Rodent; GLP-1RA differences across human populations	(181)
	PPARα agonist (PEDF)	Direct	ATGL/PPARα; HIF-1α	Promotes FAO, reduces lipid deposition, and suppresses glycolysis	Causal	Cell + Rodent; PPARα agonist selectivity	(167)
	Dasatinib + quercetin (DQ)	Direct	PPARα	Directly binds and activates PPARα, upregulates FAO-related proteins, and exerts antifibrotic effects	Causal	Cell + Rodent; DQ is a senolytic with multiple targets	(168)
	Compound H11	Direct	PPARα/PPARδ	Modulates FAO, BCAA catabolism and glycolysis, and maintains mitochondrial metabolic homeostasis	Causal	Cell + Rodent; Preclinical; dual agonist safety unknown	(169)
Chinese patent medicines	Tangshenning	Direct	Sestrin2/AMPK/PGC-1α	Regulates FAO-associated ferroptosis and restores mitochondrial homeostasis	Causal	Cell + Rodent; Complex formula; Human effects remain to be validated	(170)
	Suoquan Yishen Formula	Direct	UBC9/Drp1 SUMO1	Upregulates rate-limiting enzymes of FAO, improves lipid accumulation and renal dysfunction	Causal	Cell + Rodent; Complex formula; mechanism not fully resolved	(59)
	Yishen Huashi Granules	Indirect	Gut-kidney axis/lipid metabolic pathway	Remodels gut microbiota and may maintain lipid homeostasis partly through FAO-related pathways	Correlative	Rodent; Weak causal evidence; human-rodent microbiota differences	(182)
Lifestyle	Regular exercise	Direct	PPARδ-CPT1α; PGC-1α	Enhances FAO, improves endothelial function, and mitigates inflammation and fibrosis	Causal	Rodent; exercise protocol and intensity vary	(171, 172)
	Fasting-mimicking diet	Direct	FAO metabolic switch	Induces transient enhancement of FAO and improves metabolic flexibility	Causal	Limited human sample; safety controversy	(173, 174)
	Dietary restriction plus pharmacotherapy	Indirect	PPARα/FAO transcripts	Reduces body weight, reprograms FAO transcription, and ameliorates glomerular and tubular injury	Correlative	Rodent; Combined intervention, difficult attribution;	(183)

### 6.1 Interventions directly targeting fatty acid oxidation

#### 6.1.1 Natural compounds

Natural products often exhibit multi-target and multi-pathway characteristics and have shown therapeutic potential in DKD (159). Quercetin (QCT) is a representative active flavonoid monomer widely distributed in various edible plants, such as blueberries, Astragalus membranaceus, and Ginkgo biloba (160). Wei et al. demonstrated that quercetin upregulates fatty acid oxidation and reduces reactive oxygen species accumulation by activating the PPARA/PPARG-UCP1 signaling pathway, thereby alleviating lipid deposition and inhibiting renal fibrosis in DKD mice. These mechanisms have also been validated in *in vitro* cell experiments (161). Berberine (BBR), also known as huanglianin, is extracted from the roots, rhizomes, and tubers of plants such as Coptis chinensis, Phellodendron amurense, and Berberis soulieana, and is a typical quaternary ammonium alkaloid (162). Studies have shown that BBR promotes fatty acid oxidation by activating the PGC-1α signaling pathway, inhibits lipid deposition and excessive mitochondrial reactive oxygen species production, and restores normal mitochondrial physiological function and energy homeostasis (163). Another study confirmed that BBR can block high glucose-induced metabolic reprogramming in HK-2 cells, prevent the abnormal shift from fatty acid oxidation to glycolysis, and restore impaired mitochondrial function (164). Boeravinone C is an active component extracted from Mirabilis himalaica and has protective effects against DKD. Mechanistic studies have shown that Boeravinone C reverses high glucose-induced PPARα suppression in DKD model mice, thereby upregulating the expression of key lipid metabolism enzymes such as CPT1A and ACOX1, promoting fatty acid oxidation, and inhibiting renal tubular cell apoptosis (75). Ergosterol, a natural sterol widely present in edible fungi, has been found to improve fatty acid oxidation disorders and reduce renal tissue injury by targeting CPT1A, making it a potential active substance for the prevention and treatment of DKD (165).

#### 6.1.2 Modern drugs

PPARα is a classic target for regulating lipid metabolism and plays an important role in fatty acid oxidation (166). Pigment epithelium-derived factor (PEDF) is a PPARα agonist that promotes fatty acid oxidation by activating the ATGL/PPARα signaling pathway, significantly reduces lipid deposition in HK-2 cells, and inhibits cell differentiation. It also alleviates renal fibrosis in DKD by downregulating HIF-1α expression, inhibiting glycolysis, and regulating metabolic reprogramming (167). Dasatinib plus quercetin (DQ), a classic senolytic combination, significantly upregulates renal PPARα expression and promotes the expression of multiple fatty acid oxidation-related proteins in DKD model mice, repairing renal injury and inhibiting renal fibrosis. Molecular docking results showed that DQ can directly bind to PPARα, and knockdown of PPARα reversed the regulation of the fatty acid oxidation pathway by DQ, thereby abolishing its renoprotective effect (168). Compound H11 is a potent PPARα/PPARδ dual agonist that regulates fatty acid metabolism by activating the PPARα/PPARδ pathway, corrects lipid metabolic disorders, and also regulates branched-chain amino acid catabolism and glycolysis, participating in the homeostatic regulation of tubular mitochondrial metabolic reprogramming in DKD through multiple pathways (169).

#### 6.1.3 Chinese patent medicines

Chinese patent medicines are characterized by multi-herb compound formulas, whose multi-component synergy can exert integrated multi-target and multi-pathway regulatory effects. Tangshenning (TSN) targets the Sestrin2/AMPK/PGC-1α signaling axis to regulate fatty acid oxidation-mediated ferroptosis-related injury markers, thereby repairing renal function, reversing tubular pathological injury, and alleviating renal tissue lesions in DKD mice. *In vitro* cell experiments further verified that TSN effectively restores mitochondrial homeostasis and inhibits ferroptosis, while knockdown of Sestrin2 significantly attenuates the renoprotective effect of TSN (170). Suoquan Yishen Formula (SQYSF) inhibits SUMO E2 ligase UBC9-mediated SUMO1 modification of Drp1, upregulates the expression of key rate-limiting enzymes of fatty acid oxidation, improves lipid metabolic disorders, reduces abnormal lipid accumulation in renal tubular cells, and regulates renal dysfunction in DKD mice (59).

#### 6.1.4 Lifestyle interventions

The protective effects of regular exercise on DKD have been confirmed in multiple experimental models. In Wistar fatty rats, 8 weeks of treadmill exercise significantly inhibited albuminuria, urinary liver-type fatty acid-binding protein (L-FABP), tubulointerstitial fibrosis, inflammation, and oxidative stress (171). In SDT fatty rats, 8–16 weeks of regular treadmill training did not affect blood glucose levels but significantly improved hypertension and hyperlipidemia and prevented renal histomorphological abnormalities; exercise also upregulated renal medium-chain acyl-CoA dehydrogenase and PGC-1α expression, suggesting enhanced fatty acid metabolism. The study concluded that endurance exercise training improves DKD by ameliorating endothelial dysfunction and enhancing fatty acid metabolism, with effects independent of glycemic control (172). Regarding dietary interventions, in STZ-induced diabetic ApoE-KO mice, fasting-mimicking diet cycles were associated with reduced blood glucose and a transient metabolic switch toward enhanced FAO (173). Similarly, 6 months of periodic fasting (5-day fasting-mimicking diet or Mediterranean diet per month) induced sustained FAO enhancement, increased lipid utilization, and accelerated amino acid turnover in patients with type 2 diabetes and diabetic kidney disease (174).

### 6.2 Interventions indirectly modulating fatty acid oxidation

#### 6.2.1 Natural compounds

Stachyose is a naturally occurring functional tetrasaccharide derived from various plants such as Stachys sieboldii, Rehmannia glutinosa, and soybean. *In vitro* experiments showed that it remodels the metabolic pattern of HK-2 cells, restores fatty acid oxidation levels, and inhibits lipid accumulation by blocking abnormal glycolytic pathways. Animal experiments further confirmed that stachyose targets tyrosine 270 of SGLT2 in DKD mice and induces protein degradation, improving renal function and reducing renal fibrosis by reversing pathological metabolic reprogramming (175). Eicosapentaenoic acid (EPA) is an active component purified from fish oil that regulates fatty acid homeostasis by modulating endothelial marker CD31 expression and targets the protein kinase Cβ (PKCβ)/transforming growth factor-β (TGF-β)/plasminogen activator inhibitor-1 (PAI-1) signaling pathway to inhibit endothelial-to-mesenchymal transition (EndMT), thereby exerting renoprotective effects (176). Fucoidan is a natural active polysaccharide derived from kelp with antioxidant activity. It has been shown to improve diabetes-induced lipid metabolic disorders, reduce oxidative stress by targeting the AhR/CD36 signaling pathway, and alleviate renal injury by inhibiting fatty acid oxidation; metabolomics suggests that amino acid metabolism is a key pathway through which it exerts its effects (177). In addition, curcumin/piperine (C/P) complex effectively upregulated fecal butyrate levels and maintained intestinal and renal structural integrity in mice; *in vitro* experiments further confirmed that C/P significantly inhibits high glucose-induced renal tubular inflammation and oxidative stress injury by activating the Nrf2/HO-1 signaling pathway, thereby ameliorating renal injury (178).

#### 6.2.2 Modern drugs

Currently, the mainstream targeted drugs for clinical intervention in DKD mainly include SGLT2 inhibitors and GLP-1 receptor agonists. In addition to their glucose-lowering effects, both can indirectly regulate renal lipid homeostasis, correct abnormal fatty acid oxidation, and reduce renal lipotoxic injury. SGLT2 inhibitors have become first-line drugs for clinical intervention in DKD, and their renoprotective effects are independent of glucose lowering. Existing studies have confirmed that SGLT2 inhibitors inhibit ferroptosis, upregulate fatty acid oxidation, and alleviate renal oxidative stress injury by targeting the AMPK/NRF2/GPX4 signaling axis (179). Another study found that canagliflozin, as an SGLT2 inhibitor, also regulates amino acid metabolic reprogramming. By reversing metabolic disorders in the kidneys of DKD mice and restoring normal fatty acid oxidation, it significantly upregulates metabolic pathways such as glycine, serine, and threonine, thereby alleviating renal injury (180). Furthermore, canagliflozin corrects pathological renal metabolic reprogramming by inhibiting hypoxia-inducible factor-1α (HIF-1α) expression, reversing the abnormal metabolic shift from fatty acid oxidation to glycolysis in renal tubules (91). GLP-1 receptor agonists (GLP-1RA) have been shown to exert renoprotective effects in DKD patients. Semaglutide (Smg) is a GLP-1 receptor agonist. Studies have shown that Smg activates cyclic adenosine monophosphate (cAMP), phosphorylates protein kinase A (PKA) and cAMP response element-binding protein (CREB), significantly increases the mRNA expression of the key target β-klotho (KLB), and thereby activates the AMPK pathway, remodeling fatty acid synthesis pathways and fatty acid peroxidation antioxidant responses. Through lipid metabolic reprogramming, it inhibits ferroptosis and alleviates renal inflammation and fibrosis in DKD (181).

#### 6.2.3 Chinese patent medicines

Yishen Huashi Granules (YSHS) target the homeostasis of the “gut–kidney axis,” synchronously remodeling serum metabolites and gut microbiota, significantly increasing the abundance of Lactobacillus and Lactobacillus murinus, and reducing the abundance of Prevotella UCG_001. At the histopathological level, it alleviates glomerular mesangial matrix proliferation and tubular vacuolar degeneration, and inhibits abnormal lipid accumulation in renal tissue. Omics pathway enrichment analysis confirmed that its core effective pathway is closely related to lipid metabolism-related signaling axes, with the fatty acid oxidation pathway as a key mediating link, delaying DKD progression by regulating lipid metabolic homeostasis (182).

#### 6.2.4 Lifestyle interventions

Regarding dietary interventions, in a rat DKD model, dietary restriction combined with pharmacological treatment (fenofibrate, liraglutide, metformin, ramipril, and rosuvastatin) reduced body weight and was accompanied by improvements in glomerular and proximal tubular injury markers. The transcriptomic response was dominated by changes in peroxisomal and mitochondrial FAO transcripts regulated by fenofibrate and PPARα, localized to the proximal tubule (183). However, whether such dietary regimens are safe in diabetic patients remains controversial.

The above interventions targeting fatty acid oxidation to modulate key pathological processes of DKD have been confirmed to be effective in DKD models. However, most interventions remain at the *in vitro* and *in vivo* experimental level, with limitations such as clinical applicability and insufficient bioavailability. Future efforts should focus more on translating experimental research into clinical practice and gradually achieving clinical application.

## 7 Conclusions and perspectives

As one of the most common microvascular complications of diabetes, DKD not only seriously threatens patients’ health and markedly reduces their quality of life but also constitutes a formidable global public health challenge as its incidence continues to expand worldwide and its prevalence and mortality rise year by year. The pathogenesis of DKD is intricate, generally arising from the interplay of chronic hyperglycaemia and lipid metabolic disorder and involving the cross-regulation of multiple mechanisms. The systematic synthesis presented in this review supports a model in which defective fatty acid oxidation and mitochondrial quality control disturbance form a bidirectional self-amplifying loop embedded within a broader interaction network encompassing metabolic reprogramming, ferroptosis, ER stress, and the gut–kidney axis, rather than a linear cascade with a single upstream initiator. This offers a new theoretical anchor for understanding the heterogeneity and progression of DKD.

Nevertheless, several pressing scientific questions remain in this field. Current research is heavily concentrated on the interplay between FAO and mitochondrial function, particularly the feedback regulation within the MQC network formed by mitochondrial biogenesis, dynamics and mitophagy. Direct links between FAO and other key DKD pathological processes beyond mitochondria, such as ER stress, lysosomal dysfunction, inflammasome activation and podocyte dedifferentiation, remain sparsely investigated and are largely confined to the scope of lipid metabolism itself. Moreover, FAO is not an independent metabolic pathway but one component of lipid metabolism; although its cross-regulation with other metabolic reprogramming branches such as glycolysis and amino acid catabolism has been preliminarily described, most studies remain limited to static comparisons of individual pathways and lack systematic multipathway integration. Another noteworthy aspect is the regulation of FAO-related factors by post-translational modifications. SUMOylation promotes mitochondrial fission under DKD conditions, thereby inhibiting fatty acid oxidation; natural products targeting the SUMOylation system have demonstrated renoprotective potential (184). Acetylation of PGC-1α regulates mitochondrial biogenesis; ubiquitination is the core mechanism of PINK1/Parkin-dependent mitophagy; and lactylation of mitochondrial enzymes directly links metabolite accumulation to fatty acid oxidation flux. Whether emerging modifications such as succinylation participate in the regulation of fatty acid oxidation in DKD remains unknown.

Future studies should extend FAO research using single-cell multi-omics and spatial metabolomics. For example, combining single-cell RNA sequencing with stable-isotope tracing to map FAO metabolic flux across tubular cell populations of distinct nephron segments could precisely identify, at an early stage, the specific cell subsets most vulnerable to FAO injury together with their molecular markers, thereby advancing early intervention. At the therapeutic level, drug development targeting key FAO nodes, such as CPT1 activators, PPARα/δ dual agonists or ACOX1 modulators, has already shown potential to reduce renal lipid deposition and fibrosis in experimental models; endogenous regulatory molecules derived from the gut microbiota, such as the tryptophan metabolite IPA or short-chain fatty acids, further suggest the feasibility of restoring FAO through dietary or microecological modulation. Notably, translating these basic findings into the clinic and developing FAO-modulating strategies with cell-type specificity and no toxic side effects will be the central challenge determining whether this field can achieve clinical breakthroughs.

Existing research has established that FAO impairment participates in the core pathological progression of DKD, but most evidence remains correlational and derives mainly from cellular and animal models. Caution is therefore warranted when extrapolating these experimental conclusions to human DKD. More rigorous human studies are needed to define the causal effects of FAO deficiency in the disease course and to evaluate the translational value of related targets. Dissecting the precise regulatory network of the FAO pathway and its interactions with the metabolic pathways of the various renal cell types promises to furnish entirely new concepts and interventional strategies for the prevention and treatment of DKD.

## Data Availability

The original contributions presented in the study are included in the article/supplementary material. Further inquiries can be directed to the corresponding author.
